# The quality of life among persons with severe mental illness enrolled in an assertive community treatment program in Japan: 1-year follow-up and analyses

**DOI:** 10.1186/1745-0179-2-18

**Published:** 2006-07-31

**Authors:** Kentaro Horiuchi, Masaaki Nisihio, Iwao Oshima, Junichiro Ito, Hiroo Matsuoka, Kazumi Tsukada

**Affiliations:** 1 Konodai Hospital, National Center of Neurology and Psychiatry Japan, 1-7-1 Kohnodai, Ichikawa, Chiba, Japan; 2 Department of Mental Health and Welfare, Graduate School of Social Work, the Japan University of Social Work, 3-1-30 Takeoka, Kiyose City, Tokyo, Japan; 3 Department of Psychiatry, Tohoku University Graduate School of Medicine, 1-1 Seiryocho, Sendai, Miyagi, Japan; 4 Department of Psychiatric Rehabilitation, National Center of Neurology and Psychiatry Japan, 1-7-1 Kohnodai, Ichikawa, Chiba, Japan

## Abstract

**Background:**

Toward effective community care for persons with severe mental illness and deinstitutionalization in Japan, we assessed the impact of the first trial of an assertive community treatment program on the lives and subjective perceptions of persons with mental illness without closing hospitals.

**Methods:**

Forty-three subjects were enrolled from the newly admitted patients of a hospital, who met our criteria of problematic hospital use, severity of psychiatric disorders, and behavioral problems. The intervention team aimed to intensively support them in various life domains in their communities to decrease clients' admissions. The Quality of Life Interview was administered at baseline and after 12 months. Data were analyzed to assess the pre-post changes in their QOL, and were explained in association with other descriptive variables.

**Results:**

The objective changes included increase in persons whose longest residence in a year were in communities, increase in income, and decrease in family contacts. Most subjective items were not changed except the decrease in satisfaction with family relationships. Satisfaction with family relationships was negatively correlated with hospital days at 1 year follow-up after controlling for symptoms, but was not so at baseline. Also, correlation between satisfaction with family relationships and global well-being was attenuated. A change in the positioning of family by clients and the autonomy of clients were suggested. However, previous studies showed that dissatisfaction with family relationships predicted rehospitalizations independently from symptoms, and our findings suggest our subjects' characteristics and a possible improvement in community-based care.

**Conclusion:**

Our program predominantly fulfilled the primary goal, but it must be further refined to reflect the detailed characteristics of the target population and resource distribution. Assessing subjective perceptions, or the QOL of clients is useful for evaluating the program localization.

## Background

The rehabilitation of psychiatric patients is difficult in closed hospital settings because of institutionalism that worsens motivation and living skills [1]. And in a humane context, without mentioning the precedents in other well-developed countries, psychiatric care should be provided in environments as minimally restrictive as possible, or ideally in the community.

In Japan, we have not yet experienced psychiatric deinstitutionalization [2,3]. Against the background of rapid economic growth, private psychiatric hospitals, which account for approximately 90% of psychiatric beds, have created new wards, resulting in the highest ratio in the world of 284 beds per 100,000 persons in 1998.

The Ministry of Health, Labor and Welfare, Japan recently announced a policy for promoting the discharge of 72,000 in-patients, who were considered able for discharge if community support was prepared. Based on this, effective ways to enrich the community care for persons with severe mental illnesses were explored. To this end, we considered the assertive community treatment (ACT) model suitable because it has been well-documented and is evidence-based [4]. The ACT model is an out-reach-based psychosocial case management model that has been implemented in the United States of America for the past 30 years and has been shown to be effective in social functioning and decrease in hospital days for persons with severe mental illness [5].

Our group started the ACT-J (Assertive Community Treatment Japan) program in 2003 and organized an out-reach team that conforms to the ACT model. The team has assertively supported community living, illness management, medication, work, family relationships, and crisis handling for persons with severe mental illnesses. The primary aim of adopting the ACT model in our setting is to reduce the amount of hospital use by psychiatric high-users. A policy shared by the team was that when clients' psychotic symptoms exacerbate and when admissions are considered to have benefit, the team manages admissions. Otherwise, the team concentrates on its capacity to support clients in their communities including medical care and housing. Also, the program has an edge in that it is carefully based on clients' subjective perceptions and contractual recovery plans so that even persons with severe symptoms can have satisfying lives in the community without recurrent admissions.

However, due to flaws in the deinstitutionalization, an abundance of hospital resources, and universal health insurance coverage, not only patients and family, but also psychiatrists, albeit an inevitable choice, are liable to use admissions extensively. Even if there is no apparent worsening of psychotic symptoms, 'respite admissions' are regularly used. The merit of these admissions is their sense of ease, but the development of stress management skills in their living environments is more desirable in light of the stress-vulnerability model of schizophrenia [6].

Ideally, if we can promote their living skills and if they can achieve less stressful lifestyles, we will be able to reduce relapses and the amount of hospital uses of psychiatric high-users and thus precipitate a bed reduction. However, the impact of this intervention on clients' lives under such circumstances was unknown. In European countries, this model has been shown to be not necessarily effective according to the difference in mental health care systems [7,8]. Therefore we planned to preliminarily launch a pilot study that serves less subjects and clarify problems for dissemination.

These conditions produced a need for a comprehensive assessment of the impact on wide life domains and clients' subjective perceptions, and thus here, we mainly used an index of quality of life (QOL) that is widely used for the health care service assessment [9]. The QOL outcomes of ACT studies have shown varied results of either improved or unchanged [10]. Our hypothesis here was that, with our intervention that limits clients' psychiatric admissions to only those necessary and provides alternative support sufficiently in their communities, clients' subjective QOL would not worsen. If, contrary to our hypothesis, there were indices that were exacerbated, we tried to explain by exploring associations with other variables in order to discuss the problems.

## Methods

### Settings

The ACT-J program is operated jointly with Kohnodai Hospital, National Center of Neurology and Psychiatry, Japan, which is a relatively acute oriented hospital located in a suburban area near the capital. The program's catchment areas are the three adjacent cities whose total populations are about 1,500,000. The multi-disciplinary team was composed of 12 case managers including nurses, psychiatric social workers, psychologists, and a full-time psychiatrist at the start of the program. Its fidelity measured by the Dartmouth Assertive Community Treatment Scale (DACTS [11]) was 4.1 (excluding items related to dual diagnosis, which is not very common in Japan). The mean amount of service was approximately 2.4 hours or 4.3 contacts per month per client in the trial year, including a maximum of 8.3 hours or 19.3 times. These numbers were larger than actual feeling due to frequent phone contacts and traffic jams. Contacts included supports on self-care, housing, shopping, social skills trainings, job, illness management, medical care, and various life domains in need. The team also provides services to the subjects recruited for another randomized control study, and the total number of subjects was 77 as of August 2005.

### Subjects

Of the 922 patients newly admitted to psychiatric wards at Kohnodai Hospital since May 2003 to April 2004, 55 met the entry criteria of the program, and 43 gave informed consent to the research contents and ethical considerations such as their privacy rights. Approval of the institutional review board of Kohnodai Hospital was obtained. Subjects who did not give their consent were mostly those designated as unsuitable by hospital psychiatrists. The team met subjects before discharge and operated mainly by outreaching to the communities after discharge. The data presented hereafter are those for the 33 subjects who completed time-point semi-structured interviews at 2 weeks (baseline) and 12 months after discharge as of the end of July 2005. The other 10 subjects were excluded because they had not been discharged or one year has not passed since discharge (3 subjects), or had instability of the disease or loss of contacts (5 subjects), or was transferred to another psychiatric hospital for recuperation (1 subject), or was dead (1 subject).

The entry criteria were as follows: Age 18–59. Resident in one of the three cities in the catchment area. Diagnosed as having severe mental illness such as schizophrenia or related disorders, or major mood disorders by ICD-10 [12] at the time of admission. Additionally, the subject must have had frequent admissions or uses of the emergency unit or had non-adherence to psychiatric care, and have problematic behavior such as violence, substance misuse, disappearance, homelessness, suicide attempts, and have not had constant social roles or the ability for self-care in the previous two years. Persons with a main diagnosis of mental retardation, dementia, substance use disorders, or personality disorders were excluded. Also excluded were patients of brief admissions focused on care for drug addiction or patients limited to physical treatment mainly for those who had been hospitalized in other psychiatric hospitals.

Table 1 shows the socio-demographic and psychiatric characteristics of the subjects. The mean age was 33.9 (SD 10.2, range 19–57). The mean hospital days during the year before the index admission were 105.8 (SD 87.7, range 0–351), the mean number of admissions was 1.6 (SD 1.4, range 0–6), with persons who had discontinuance of treatment and who were mostly hospitalized for a full year.

**Table 1:** Characteristics of the subjects

		(N = 33)
Age (mean ± SD)		33.9 ± 10.2
Sex	Male : Female	14 (42.4%): 19 (57.6%)
Psychiatric diagnoses	Schizophrenia and related disorders	22 (67.7%)
		
(ICD-10)	Mood disorders and related disorders	6 (18.2%)
	Others	5 (15.1%)
Years of Education (mean ± SD)		12.4 ± 1.9
Marriage (number (%))		4 (12.1%)
Hospital days in the previous year (mean ± SD)		105.8 ± 87.7
Number of admissions (mean ± SD)		1.6 ± 1.4
Years of illness (mean ± SD)		10.9 ± 7.4

As for diagnosis, schizophrenia and related disorders (ICD-10: F2x) were the most frequent (67.7%), and mood disorders (F3x, 18.2%) and others (15.1%) comprised the remainder. Among these characteristic data, significant correlations between age and duration of illness (r = .607, *P *< .001), and between the number of admissions and hospital days (r = .479, *P *= .005) were found. Between subjects that dropped out and that were followed-up, the former were older (t = 2.67, *P *= .011, df = 40) and had a longer duration of illness (t = 4.31, *P *< .001, df = 40) than the latter.

### Procedures

We obtained socio-demographic data and information on hospital use from medical records. The Quality of Life Interview (QOLI) [13] was administered at 2 weeks (baseline) and 12 months after discharge of the index admission. QOLI is a comprehensive questionnaire to assess subjects' objective living situations and subjective life satisfaction. The Japanese translated version of QOLI had been validated beforehand by Oka et al. (unpublished) The data at baseline and 12 months were compared using the Wilcoxon signed rank tests or binomial tests as appropriate.

Other variables were used for explanations mainly by correlation analyses. These included hospital days, the Brief Psychiatric Rating Scale (BPRS) [14] for two time points as well as QOLI, and the amount of services provided by our team calculated from the service log of the team. A Japanese translation of BPRS was provided by Kitamura et al. Statistic calculations were performed by the Statistical Package for Social Sciences (SPSS) version 10.0 (SPSS Inc.).

## Results

Table 2 shows changes in psychiatric symptoms (BPRS) and hospital days before and after interventions. No significant change in total score of BPRS (Z = -0.76, *P *= .445) were found, while hospital days significantly decreased (Z = -3.55, *P *= .000). There were no significant correlations between BPRS and hospital days at baseline and 12-month either.

**Table 2:** Psychiatric Symptoms and hospital days before and after interventions

	*Baseline*		*12 months later*			
				
	mean	SD	mean	SD	Z	p
Total score of BPRS	15.7	6.2	16.5	7.2	-0.76	.445
Hospital days	105.8	87.7	34.9	65.3	-3.55	**.000**

Table 3 shows a comparison between the baseline and 12-month scores of the QOLI. On objective QOL, in the domain of housing, the number of subjects whose longest residency in the previous year was in communities increased (binomial test against baseline data, *P *= .004). Even if not statistically significant, those living by themselves increased from 6 (18.2%) to 10 (30.3%), with no large change in the number living with their families. The frequency of family contact decreased significantly (Z = -2.05, *P *= .041). From the financial perspective, although there are some missing data, the increase in income was significant (Z = -3.33, *P *= .001).

**Table 3:** Pre-post comparisons of QOL items

	*Baseline*		*12 months later*			
				
*Subjective items*	Mean	SD	Mean	SD	Z	*P*
Housing	4.46	1.47	4.27	1.43	-0.87	.384
Leisure activities	3.75	1.49	3.95	1.31	-0.60	.550
Family relationships	4.45	1.94	3.76	1.62	-2.04	**.042**
Social relationships	3.90	1.51	3.89	1.55	-0.03	.974
Finances	3.93	1.55	3.70	1.46	-0.73	.468
Safety	4.70	1.49	4.10	1.56	-1.73	.083
Health	3.54	1.18	3.38	1.48	-0.59	.557
Global well-being	4.09	1.64	4.03	1.66	-0.07	.945
						
*Objective items*						
The longest residence was in communities (No. of persons (%))*	25	75.8%	32	97.0%		**.004**
Frequency of family contacts	4.27	1.33	3.91	1.55	-2.05	**.041**
Income†	74.4	49.4	103.6	61.9	-3.33	**.001**

*Binomial test, one-tailed *P*.

† df = 21

As for subjective QOL, the score of global well-being did not change (Z = .007, *P *= .945). The characteristics of the subjects did not affect global well-being. For each life domain of satisfaction, only satisfaction with family relationships significantly decreased. As for the relation between satisfaction with family relationships and the characteristics of subjects, age negatively correlated with satisfaction (r = -.446, *P *= .009) at baseline; but not after 12 months (r = .111, *P *= .538). Age was positively correlated with the change in satisfaction with family relationships (r = .528, *P *= .002). Satisfaction with life domains other than family relationships also negatively correlated with the age of the subjects at baseline, but it did not correlate with age after 12 months.

A significant correlation was found between satisfaction with family relationships and global well-being at the baseline, but not at 12 months after (Table 4). Satisfaction with family relationships did not differ by whether clients lived with families or not (Mann-Whitney test; Z = -0.54, *P *= .592 for baseline; Z = -0.60, *P *= .550 for 12 months later).

**Table 4:** Correlations between subjective QOL in life domains and global well-being

	*Baseline*		*12 months later*	
		
	r	*P*	r	*P*
Housing	.759	.000	.684	.000
Leisure activity	.831	.000	.625	.000
Family relationship	.605	.000	.311	.078
Social relationship	.572	.001	.649	.000
Finances	.574	.001	.287	.111
Safety	.474	.005	.277	.124
Health	.114	.526	.330	.065

When exploring variables correlated with satisfaction with family relationships (Table 5), significant negative correlation with the amount of hospital days in the previous year was found (r = -.432, *P *= .012), but not at the baseline (r = -.042. *P *= .815). This relationship was significant after controlling symptoms. Another variable which correlated with satisfaction with family relationships was the frequency of planned events with someone other than the family (r = .375, *P *= .031 for baseline; r = .578, *P *= .001 for 12 months later) and this correlation was also significant after controlling symptoms (r = .339, *P *= .058 for baseline; r = .529, *P *= .002 for 12 months later). Neither satisfaction with family relationships (r = -.280, *P *= .115) nor the global well-being (r = -.233, *P *= .192) were correlated with symptoms at after 12 months. The amount of service by the team also correlated with satisfaction at 12 months later (r = .445, *P *= .010).

**Table 5:** Correlations with hospital days in the preceding year

		*Baseline*		*12 months later*	
			
		r	2-tailed *P*	r	2-tailed *P*
Satisfaction with family relationships	*Simple correlation*	-.042	.815	-.432	**.012**
	Partial correlation*	.178	.330	-.454	**.009**
Global well-being	*Simple correlation*	.081	.654	-.114	.528

*Controlling for the sum of BPRS scores.

## Discussion

### Pre-post comparisons

Subjects who stay longest in the communities increased, their incomes increased, their family contacts decreased, significant reduction in hospital days, and no significant changes in psychiatric symptoms among the subjects. The primary aim of this intervention, that is, to provide alternative assertive support in order to limit unnecessary admissions, seems to have being fulfilled through substituting the family burden. Moreover, clients' subjective QOL in most life domains and global well-being were not decreased and our hypothesis was partly supported. However, solely deteriorated was satisfaction with family relationships, and this was regarded important because it was reported that it predicted rehospitalizations independently from the severity of symptoms and the previous rehospitalization [15]. It was assumed that satisfaction with family relationships indicates an unmet need for care among this population.

### Association between satisfaction with family relationships and admissions

Persons who reported low satisfaction with family relationships were those who used more admissions. Presumable causes from observations were clients' friction with their families, 'respite admissions' co-occurring with burn-out of their families, neurotic symptoms related to anxiety among clients who live by themselves, and preventive admissions consensual among psychiatrists, clients and families.

At baseline, decisions on discharge were made by hospital psychiatrists, while the team intervened in the decision after the enrollment. Previous study showed the decision on discharge was generally made in view of not only psychiatric symptoms, but also the prospects for continuing community living [16], and thus, at baseline, their dissatisfaction with family relationships might be one of the factors. On the other hand, for admissions under commitment by the team, the prospects for continuing community living might increase more than usual with the flexible support provided by the team, and then the decision might be more dependent on the severity of symptoms.

This might have aroused clients' dissatisfaction, but this was ambivalent because this might also have led them to an increased use of community resources instead of being stuck between family and hospital.

### Program modifications suggested by QOL

Our result showed that the correlation between satisfaction with family relationships and global well-being was significant at baseline but not at one year later. This could imply the change in the positioning of family by them. A previous study in Japan reported lower QOL in persons with mental illness who live with their families than in those who do not after controlling symptoms [17]. Considering our data showed that satisfaction with family relationships was not associated with whether they lived with families or not, our approach to this issue was successful.

However, our results suggest a few points that must be redeemed. Without the deinstitutionalization, we could not have obtained evident information on the characteristics of these high-users in our country. It was reported that the ACT program could reduce in-patient care but at the expense of increasing social dysfunction and behavioral disturbance for persons with dual diagnoses of psychotic disorders and personality disorders [18], and was perhaps not sufficiently effective in persons who have mental illness and intellectual disability [19]. We used one main diagnosis, but subjects possibly included those with duality. This must be reviewed and we should provide more suitable services. In relation to this, we sometimes found that clients or families had strong dependency on hospitalization. Admitting this was expected in a situation lacking experiences of systematic community-based care, the team required much effort to motivate stakeholders when exploring alternative solutions with clients in their communities. It was reported that degree of sick-role is related to frequency and duration of admission [20], while researches on the scheduled intermittent admission have shown positive outcomes [21]. Although this problem does not have one optimal solution, we should cooperate more with hospital staff as well as clients and families on evaluating how much utilization of hospital resources would be appropriate.

### Limitations and significations

Because this research was strictly targeted and thus the sample size was small, statistical analyses were inevitably limited. Furthermore, 10 subjects among 43 enrollees were excluded in priority to clinical reasons, therefore results here were provisional in this regard. Subjects who dropped out were older and age was correlated positively with the change in QOL, so we couldn't presume these biased results positively, though, in consideration of one subject who died at the beginning of enrollment, we must interpret our result more cautiously.

Having adopted an evidence-based intervention in a new setting and having analyzed the change in the comprehensive QOL allowed us to clarify problems to refine our program and contribute to knowledge of the characteristics and a possible community treatment for high-users of psychiatric hospitals. In the future, we shall conduct subsequent researches including qualitative approaches and social costs.

## Conclusion

The first systematic replication of the ACT program in Japan is fulfilling its initial aim to adjust hospital use of psychiatric high-users by providing them with necessary alternative support in their communities. Clients report mostly unchanged subjective QOL and the hypothesis of this research was partly supported; however, satisfaction with family relationships specifically declined. Under the intervention, satisfaction with family relationships correlated with the amount of hospital days and it was suggested, although ambivalently, that limited use of admission alone might be insufficient for some people in this setting in terms of satisfaction with family relationships. We must improve our program to deal with this need by confirming the target population and assembling knowledge of their genuine need for hospitalization. Researches on comprehensive QOL and clients' perceptions should be accumulated in order to reform the mental health care system safely.

## Competing interests

The author(s) declare that they have no competing interests.

## Authors' contributions

KH planned research, acquired data, analysed, interpreted data, and drafted the manuscript. NM acquired and interpreted data and helped to draft the manuscript. IO designed the research project and acquired service process data. JI designed the research project and made revisions mainly to family relationships. HM made revisions mainly to the medical aspects. KT managed the research project and made critical revisions. All the authors read and approved the final manuscript.
